# Pharmacogenetics–Based Preliminary Algorithm to Predict the Incidence of Infection in Patients Receiving Cytotoxic Chemotherapy for Hematological Malignancies: A Discovery Cohort

**DOI:** 10.3389/fphar.2021.602676

**Published:** 2021-03-10

**Authors:** Matias F. Martinez, Enzo Alveal, Tomas G. Soto, Eva I. Bustamante, Fernanda Ávila, Shrikant I. Bangdiwala, Ivonne Flores, Claudia Monterrosa, Ricardo Morales, Nelson M. Varela, Alison E. Fohner, Luis A. Quiñones

**Affiliations:** ^1^ Laboratory of Chemical Carcinogenesis and Pharmacogenetics (CQF), Department of Basic and Clinical Oncology (DOBC), Faculty of Medicine, University of Chile, Santiago, Chile; ^2^ Departamento de Ciencias y Tecnología Farmacéuticas, Facultad de Ciencias Químicas y Farmacéuticas, Universidad de Chile, Santiago de Chile, Chile; ^3^ Latin American Network for the Implementation and Validation of Pharmacogenomic Clinical Guidelines (RELIVAF-CYTED), Madrid, Spain; ^4^ Departamento De Ciencias Básicas Santiago, Facultad De Ciencias, Universidad Santo Tomás, Santiago, Chile; ^5^ Cancer Institute Arturo López Pérez Foundation, Santiago, Chile; ^6^ Clinical Hospital of the University of Chile, Santiago, Chile; ^7^ Population Health Research Institute, McMaster University, Hamilton, ON, Canada; ^8^ Department of Health Research Methods, Evidence, and Impact, McMaster University, Hamilton, ON, Canada; ^9^ Department of Epidemiology and Institute of Public Health Genetics, University of Washington, Seattle, WA, United States

**Keywords:** pharmacogenetics, hematological malignancies, infections, Prediction, Algorithm, pharmacogenomics, CYP3A4, OAT4

## Abstract

**Introduction:** Infections in hematological cancer patients are common and usually life-threatening; avoiding them could decrease morbidity, mortality, and cost. Genes associated with antineoplastics’ pharmacokinetics or with the immune/inflammatory response could explain variability in infection occurrence.

**Objective:** To build a pharmacogenetic-based algorithm to predict the incidence of infections in patients undergoing cytotoxic chemotherapy.

**Methods:** Prospective cohort study in adult patients receiving cytotoxic chemotherapy to treat leukemia, lymphoma, or myeloma in two hospitals in Santiago, Chile. We constructed the predictive model using logistic regression. We assessed thirteen genetic polymorphisms (including nine pharmacokinetic—related genes and four inflammatory response-related genes) and sociodemographic/clinical variables to be incorporated into the model. The model’s calibration and discrimination were used to compare models; they were assessed by the Hosmer-Lemeshow goodness-of-fit test and area under the ROC curve, respectively, in association with Pseudo-R^2^.

**Results:** We analyzed 203 chemotherapy cycles in 50 patients (47.8 ± 16.1 years; 56% women), including 13 (26%) with acute lymphoblastic and 12 (24%) with myeloblastic leukemia.

Pharmacokinetics-related polymorphisms incorporated into the model were *CYP3A4* rs2242480C>T and *OAT4* rs11231809T>A. Immune/inflammatory response-related polymorphisms were *TLR2* rs4696480T>A and *IL-6* rs1800796C>G. Clinical/demographic variables incorporated into the model were chemotherapy type and cycle, diagnosis, days in neutropenia, age, and sex. The Pseudo-R^2^ was 0.56, the *p*-value of the Hosmer-Lemeshow test was 0.98, showing good goodness-of-fit, and the area under the ROC curve was 0.93, showing good diagnostic accuracy.

**Conclusions:** Genetics can help to predict infections in patients undergoing chemotherapy. This algorithm should be validated and could be used to save lives, decrease economic costs, and optimize limited health resources.

## Introduction

Infections in patients diagnosed with hematological malignancies are common and usually life-threatening (Green 2017); 30% of patients undergoing chemotherapy for the treatment of these illnesses experience infection, and 11% dying as a result (Castagnola et al., 2007; Yilmaz et al., 2008; Dutronc et al., 2009). Several variables have been described as risk or protector factors. In addition to clinical variables associated with a high risk of infection, neutropenia, defined as an absolute neutrophil count lower than 500 cells/mm^3^ (Klastersky et al., 2016), is common in patients undergoing cytotoxic chemotherapy (Li et al., 2016). Genetics can modify the occurrence of chemotherapy-related neutropenia. A polymorphic variant can alter the metabolism or elimination of the cytotoxic agent; this could increase the plasmatic level of the antineoplastic, and therefore increase the risk of dose-related toxicity (Lyman et al., 2014; Buaboonnam et al., 2019). Among these pharmacokinetics-related genetic factors, CYP3A4 and CYP3A5 are two enzymes that participate in the metabolism of most drugs used in the treatment of hematological malignancies (Lee et al., 2013; Daly 2015), and polymorphic variants in the genes that encode those proteins are known to decrease their expression or functionality, decreasing drug metabolism (Lamba et al., 2012; Park et al., 2014; Zhu et al., 2014). Moreover, polymorphic variants in genes that codify for drug transporters could affect the elimination of antineoplastic medications (Choi et al., 2015). Some of the transporters associated with cancer medications are ATP-Binding Cassette (ABC), specifically ABCB1, ABCC2, and ABCG2 (Jedlitschky et al., 2006; Vasiliou et al., 2009), and Solute Linking Carrier (SLC), specifically *SLC22A11* that codifies for the Organic Anion Transporter 4 (OAT4) (Burckhardt 2012). Genetic variants in these genes could increase the risk of adverse events due to decreased elimination leading to toxic drug concentrations (Vormfelde et al., 2006; Lewis et al., 2013).

Furthermore, some immune/inflammatory response-related proteins can improve the aggregation and survival of neutrophils. Interleukin 6 and 1β (IL6 and IL-1β) participate in the maturation and apoptosis inhibition of white blood cells (WBC) (Rose-John et al., 2017; Chiba et al., 2018). A lower expression of the genes that encode these interleukins could enhance the risk of neutropenia, make it more severe or prolong the length of neutropenia (Wright et al., 2014; Loft et al., 2018; Badawy et al., 2019). The caspase recruitment domain 8 (CARD8) participates in the activation of IL-1β, and a polymorphism that affects CARD8 functionality could have the same consequences of having less interleukin signal (Paramel et al., 2015). Toll-like receptor 2 (TLR2) is a protein that senses pathogen molecules and develops the intracellular signaling in response to a possible infection (Beutler et al., 2006); a genetic variant that affects the functionality of this receptor could increase the risk of infections (Hawn et al., 2009; Bielinski et al., 2011; Esposito et al., 2014).

No tool can predict the incidence of infections in hematological cancer patients in chemotherapy that use clinical and genetic variables. By accurately predicting which patients are likely to develop an infection while undergoing chemotherapy, dose adjustments and enhanced monitoring could be targeted to prevent infection, thereby reducing morbidity, mortality, and healthcare costs. Our study aims to create an algorithm to predict the incidence of infections among patients undergoing chemotherapy, using pharmacokinetics and immune response-related genetic polymorphisms in addition to clinical variables.

## Materials and Methods

### Study Design

We carried out a prospective cohort study from November 2017 to October 2018 at the Oncologic Hospital “Fundación Arturo Lopez Pérez” (FALP) and the Clinical Hospital of the University of Chile (HCUCH) in Santiago, Chile. Patients were enrolled at those clinical centers before the first chemotherapy cycle, and they were followed prospectively through every cycle of chemotherapy. Infection was established by clinical criteria, including fever not explained by chemotherapy, positive bacterial cultures, or compatible imaging. A multidisciplinary team compound by specialist physicians and clinical pharmacists reviewed every febrile episode and decided if it was due to infection or not according to local practice. The occurrence of any infections was recorded.

### Patients and Data Source

The study included patients 18 years or older diagnosed with leukemia or non-Hodgkin’s lymphoma and undergoing cytotoxic chemotherapy. We excluded patients regularly taking immunosuppressive medication, pregnant women, and patients with a diagnosis of immunodeficiency. All clinical data were obtained from the medical record. We collect the information that allows clinic, pharmacotherapeutic, morbid, and demographic characterization of the sample.

### Ethics Statement

All patients signed a written informed consent and an agreement to participate in this study. The study was carried out following the strict ethical procedures recommended by the Ethics Committee of the Clinical Hospital of the University of Chile (approval received on July 18, 2017) and the Eastern Metropolitan Health Service (approval received on July 4, 2017), following the procedures suggested in the Declaration of Helsinki, with Chilean Laws 20.120, 20.584, and 19.628 and with the guidelines of the Good Clinical Practices from the World Health Organization.

### Genotyping Analysis

Genomic DNA was isolated from the subjects’ peripheral blood samples using the High Pure PCR Template Preparation Kit (Catalog Number, 11796828001; Roche Diagnostics GmbH, Mannheim, Germany). The blood sample was collected after the first remission.

The nine pharmacokinetics-related polymorphisms were *SLC22A11* rs11231809; *ABCB1* rs2032582, rs1045642y rs1128503; *CYP3A4* rs2740574, rs2242480, *CYP3A5* rs15524, *ABCC2* rs12762549, and *ABCG2* rs2231142. The immune response-related polymorphisms were *IL6* rs1800796, *IL1*β rs1143627, *CARD8* rs2043211, and *TLR2* rs4696480. The potential effect in protein was the main criteria to pick the polymorphic variants in addition to the relationship with drug toxicity reported in the literature. Another factor considered was the minor allele frequency of more than 5% in the Latinamerican population when available.

All polymorphisms were analyzed using *TaqMan* ^®^ SNP Genotyping Assay (Catalog number, 4362691; Thermo Fisher Scientific, Waltham, MA, United States) in a Stratagene Mx3000p real-time PCR system (Agilent Technologies, Santa Clara, CA, United States). Every sample was analyzed in triplicate to ensure reliability. The datasets presented in this study can be found in online repositories. The names of the repository/repositories and accession number(s) can be found below: [10.6084/m9.figshare.13444211].

### Statistical Analysis

To determine which genetic variants were associated with infection incidence, it was a binary variable, and it was assessed in every chemotherapy cycle. We carried out multivariate logistic regression models to establish the relationship between every polymorphism by itself and the outcome. We assessed the inheritance in a dominant, recessive, and co-dominant model, and they were added depending on their statistical significance. These models included one genetic variable and the following control variables: sex; age (in groups: from 18 to 40 years; from 41 to 60 years and older than 60 years); the number of chemotherapy cycle; kind of chemotherapy scheme (induction or consolidation), diagnosis (acute lymphoblastic leukemia, acute myeloblastic leukemia, other leukemias or lymphoma) and the number of days in profound neutropenia, defined as an absolute neutrophil count of 0 cells/mm^3^.

Adjusted Odds Ratios (OR) were obtained from exponentiating the coefficient given by the regression model. The ORs were used to determine which variables were risk or protective factors of having an infection.

To develop the final model, we added all the control variables and those genetic factors that had a statically significant association with infection in the previous step. The best model was chosen according to the value of Pseudo-R^2^, the calibration, and the discrimination of the model. The control variables were chosen based on the univariate relationship or previous reports of the association’s association with the event.

We use a mixed-effect model to account for intraindividual variability regarding chemotherapy scheme and cycle and use correlated outcome data (also known as hierarchical models). Here we used a three-level model, so we had a random intercept for three characteristics. The first one was the patient level, the second chemotherapy scheme, and finally cycle level, where infections were assessed.

Calibration is the degree of similarity between the probability given by the model and the observed incidence. The goodness-of-fit test of Hosmer—Lemeshow compares frequencies of cases and controls using a chi2 test. In this case, a higher *p*-value indicates fewer differences between the predicted frequencies and the frequencies observed in the sample.

On the other hand, discrimination is the degree to which the proposed model can distinguish between patients who experience the event from those who do not; that is, the ability to indicate that a patient will experience an infection, and, on the other hand, the ability to predict when the patient will not experience it. Discrimination between cases and non-cases of infection was tested using the area under the Receiver Operating Characteristic curve (AUC-ROC).

The sample characteristics were mainly presented as proportions. The Shapiro–Wilk test was used to assess the normality of the distribution of continuous variables, and when the distribution was skewed, the variable was presented as median and interquartile range. All the analyses and figures were performed using STATA 15.0 software ®.

## Results

### Sample Characteristics

Participants’ median age was 40 years (IQR 31–52), and 27 (54.0%) were women. Half of the patients had acute leukemia [13 (26%) lymphoblastic and 12 (24%) myeloblastic] (Table 1). We detected infection in 82 (40.8%) of 203 total chemotherapy cycles, including 31(62.0%) of the 50 patients recruited. No patients died during the follow-up period. The genotype distribution of the thirteen polymorphisms assessed in the study is given in Table 2.

**TABLE 1:** Sample characteristics.

Characteristics	n = 50 (%)
Age, years (median ± IQR)	40 (31–52)
18–40 years	17 (34)
41–60 years	20 (40)
>60 years	13 (26)
Female	27 (54)
Male	23 (46)
Diagnosis	
Acute lymphoblastic leukemia	13 (26)
Acute myeloblastic leukemia	12 (24)
Other leukemias	5 (10)
Lymphoma	20 (40)

**TABLE 2:** Allele and genotypic frequencies of the studied polymorphism.

Genetic polymorphism	Allele frequency		Genotypic frequency, n (%)		
CARD8 rs2043211	A	T	A/A	A/T	T/T
	0,64	0,36	19 (39)	25 (51)	5 (10)
TLR2 rs4696480	T	A	T/T	T/A	A/A
	0,63	0,38	20 (42)	20 (42)	8 (17)
IL-6 rs1800796	G	C	G/G	G/C	C/C
	0,50	0,50	11 (22)	28 (56)	11 (22)
IL-1β rs1143627	G	A	G/G	G/A	A/A
	0,54	0,46	16 (33)	21 (43)	12 (24)
OAT4 rs11231809	T	A	T/T	T/A	A/A
	0,38	0,62	11 (24)	13 (28)	22 (48)
ABCB1 rs2032582	C	A	C/C	C/A	A/A
	0,68	0,32	21 (53)	12 (30)	7 (18)
ABCB1 rs1045642	A	G	AA	AG	GG
	0,33	0,67	3 (6)	27 (54)	20 (40)
ABCB1 rs1128503	A	G	AA	AG	GG
	0,38	0,62	7 (14)	24 (48)	19 (38)
CYP3A4 rs2740574	T	C	T/T	T/C	C/C
	0,83	0,17	31 (74)	8 (19)	3 (7)
CYP3A4 rs2242480	C	T	CC	CT	TT
	0,71	0,29	29 (59)	12 (24)	8 (16)
CYP3A5 rs15524	A	G	AA	AG	GG
	0,84	0,16	37 (74)	10 (20)	3 (6)
ABCC2 rs12762549	G	C	G/G	G/C	C/C
	0,62	0,38	19 (42)	18 (40)	8 (18)
ABCG2 rs2231142	G	T	G/G	G/T	T/T
	0,90	0,10	37 (80)	9 (20)	(0)

Some samples could not be fully genotyped.

## Construction of the Predictive Model

### Model With Non-genetic Variables

To assess how genetics can improve the predictive model’s performance, we carried out primarily a non-genetic algorithm. For this, we incorporated chemotherapy type and cycle, diagnosis, days in neutropenia, age, and sex. The *p*-value of the Hosmer – Lemeshow test was 0.29, the AUC-ROC was 0.82, and the Pseudo-R^2^ was 0.23.

### Model With Genetic Variables

We found that Toll-Like Receptor 2 (*TLR2*), Interleukin 6 (*IL6*), CYP3A4, Solute Linking Carrier family 22 member 11 (*SLC22A11*) or Organic Anion Transporter 4 (OAT4), and ATP-Binding Cassette Subfamily C member 2 (*ABCC2*) polymorphisms were associated with the incidence of infection by themselves (Supplementary Table S1), and they were added to the final model.

**FIGURE 1:**
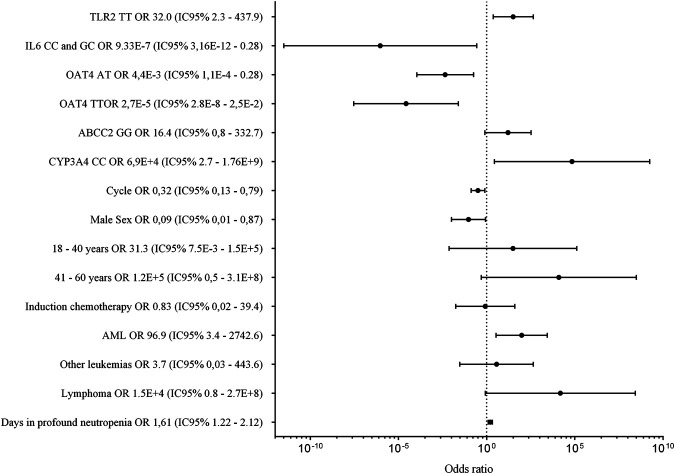
Genetic and non-genetic factors associated with infection in patients treated with cytotoxic chemotherapy. The figure presents the point estimate of the odds ratio for infection, and the bars around each point show the 95% confidence interval.

In Figure 1, we summarize the OR obtained from the adjusted final model. There we found that TLR2, IL6, OAT4, and CYP3A4 were significantly associated with the occurrence of infection. The Pseudo R2 of the model was 0.5327; i.e., the model explained 53% of the variability of the incidence of infections.

The multilevel model with a random intercept for patient, chemotherapy scheme, and cycle variables gave the same calibration and discrimination performance. As we had a predictive objective and allowing a more straightforward application in clinical practice, we construct the model only with fixed intercepts, i.e., a “normal” logistic regression.

### Calibration of the Model

The *p*-value of the test of Hosmer—Lemeshow was 0.9516; this means that the model had an excellent goodness-of-fit or, in other words, there is no statistical difference between the frequency of cases detected by the model and that observed in the sample.

### Discrimination of the Model

The AUC-ROC indicates the probability of assigning a higher probability of incidence to patients who develop an infection than those who do not. The ROC curve compares sensitivity, that is, the ability to assign the event correctly when it occurred, and one- specificity, that is, the proportion of patients who did not have an infection, but the model indicated that they would present it.

**FIGURE 2:**
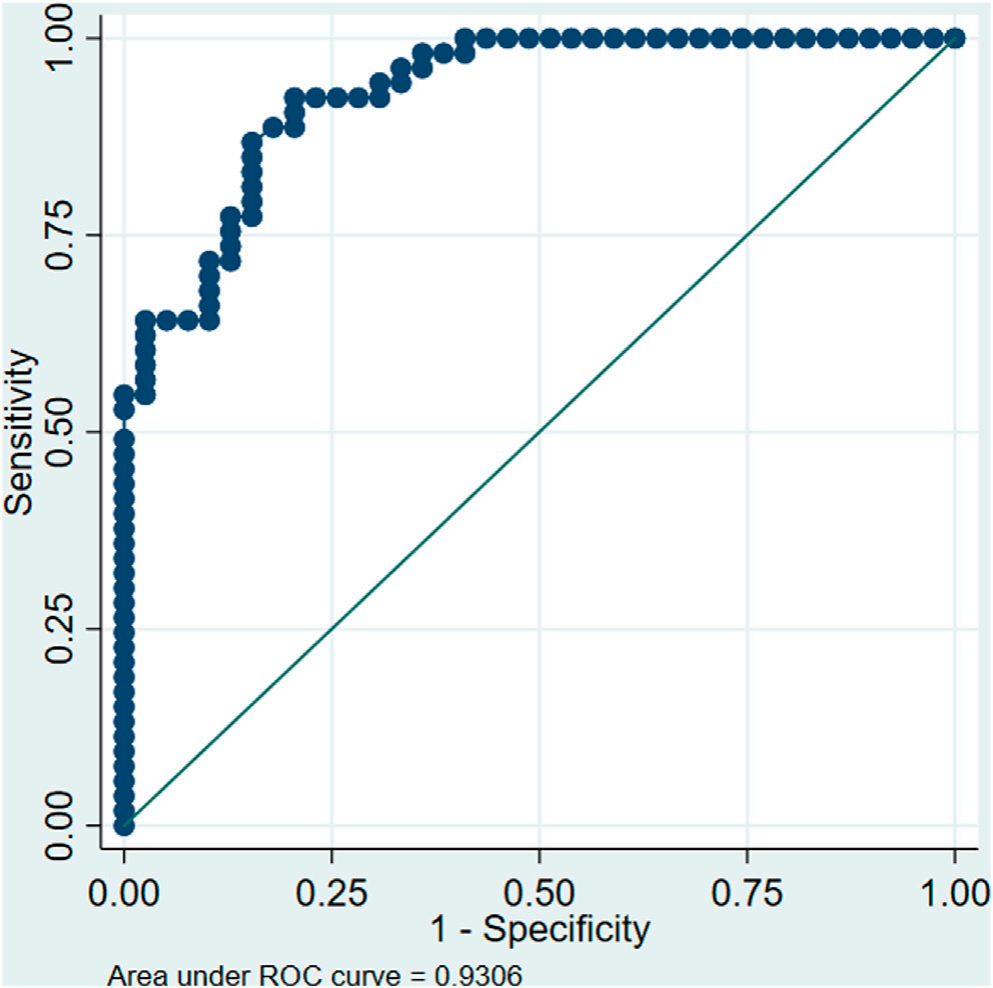
Receiver Operating Characteristic (ROC) curve for the model proposed to predict infections in patients undergoing cytotoxic chemotherapy. (References TLR2 TA/AA, IL6 GG, OAT4 AA, ABCC2 CC/CG, CYP3A4 TT and CT, Female sex, 60 years or older, Consolidation chemotherapy, Acute Lymphoblastic Leukemia, respectively for each category).

The AUC-ROC curve can range from 0 to 1, with 0.5 indicating that the instrument cannot discriminate outcomes better than chance. A value of one would be for a model that perfectly predicts the occurrence of the event. For our proposed model, the AUC-ROC curve was 0.93. Figure 2 shows the ROC curve for the model to predict the incidence of infections in the sample. Concerning non-genetic variables, we found that the number of chemotherapy cycles was a protective factor. This result could be interpreted as follows: with each cycle of chemotherapy that passes, the risk of infection decreases, perhaps because if the patient had an infection in an initial cycle, in the following cycles, the prophylaxis would be optimized to avoid futures events. Besides, the male sex was protective compared to the female sex; induction was associated with more risk than consolidation, and the longer neutropenia episodes were associated with more risk of infection.

The coefficients for every variable obtained from the final regression model allow us to build an equation to predict the probability of infection occurrence. Thus, the model, including genetic and non-genetic variables, is:

$$\begin{array}{l} \ln\left(\frac{p}{1-p}\right)=3.47\times \left(TLR2\;TT\right)-\;13.9\times \left(IL6\;GG\right)5.4\times \left(OAT4\;AT\right)-10.5\;\times \;\left(OAT4\;TT\right)+\;\\ \;2.8\times \left(ABCC2\;GG\right)+\;11.1\times \left(CYP3A4\;CC\right)-1.1\times \left(Cycle\right)-2.4\times \left(Male\;sex\right)+3.4\;\times \;\\ \left(From\;18\;to\;40\;years\right)+9.4\times \left(From\;41\;to\;60\;years\right)+0.2\;\times \;\left(Induction\right)+4.6\times \left(AML\right)+\\ 1.3\times \left(Other\;leukemias\right)+9.6\times \left(Lymp\mathrm{hom}a\right)+0.5\;\times \;\left(Days\;in\;deep\;neutropenia\right)-1.9 \end{array}$$

where *p* is the probability of occurrence of infection, with values ranging from 0 to 1 (or 0%–100% of occurrence probability). Some factors increased the odds of having an infection (positive factors), and others decreased the odds of the event (negative factor), according to the OR.

For genetics, the model works with dichotomous variables (i.e., 0 and 1). Thus when the variant showed in the model is present, we should use a 1, and when it is another genotype, we should use a 0. For example, in the OAT variant, if the genotype TT were determined, the coefficient for OAT AT would be multiplied by 0 (5.4 × 0) because the patient presents another genotype, and the coefficient for TT genotype should be multiplied by 1 (10.5 × 1). Alternatively, if the genotype AA were determined, the coefficients for TT and AT should be multiplied by 0. Days in neutropenia should be used as a continuous variable. Meanwhile, the days in neutropenia increase, also the risk of infection does.

## Discussion

Infections are frequent and potentially lethal events in patients treated with cytotoxic chemotherapy for hematological malignancies. Neutropenia due to the medication and the illness by itself is common in these patients, and it is one of the more critical factors in the incidence of infections. This study is the first that aims to predict their occurrence using genes associated with both antineoplastic pharmacokinetics and immune response.

### Relationship Between Genetics and the Incidence of Infections

Concerning immune response-related genes, we found two polymorphisms significantly associated with infections. One of them was *IL6* rs1800796 (−572C>G), which is a variant located in the promoter of the gene and where the G allele is related to a lower expression of the protein (Sharma et al., 2018). We found that the GG genotype was a risk factor for having an infection, agreeing with previous reports (Tang et al., 2014; Badawy et al., 2019), probably due to a lower neutrophil mobilization and activation (Wright et al., 2014; Rose-John et al., 2017).

The other immune response-related polymorphism associated with infections was *TLR2* rs4696480 (c.-373+1614T>A). Although the variant’s effect at the protein level is not well established, the T allele has been associated with lowering receptor functionality (Loft et al., 2018), a decreased function of *TLR2* prevents the pathogen recognition and delay the immune response (Hawn et al., 2009). We found the TT genotype was a risk factor of having an infection compared to TA and AA genotypes as we expected because the *TLR2* receptor also promotes neutrophil recruitment and survival (Malcolm et al., 2003; Sabroe et al., 2005).

Concerning pharmacokinetics–related polymorphisms, *SLC22A11* rs11231809 polymorphism was associated with the risk of infection. We found that the genotype TT and AT are protector factors compared to the AA genotype. This finding is consistent with previous reports describing that the A allele is associated with a lower functionality of the protein OAT4. This variant has been associated with a decreased clearance of some drugs (Vormfelde et al., 2006; Lima et al., 2015), so perhaps a lower elimination leads to a higher plasmatic level of antineoplastic that causes dose-related toxicities.

We found the polymorphic variant rs2242480 in the gene *CYP3A4* that encodes the biotransformation enzyme CYP3A4 was related to the incidence of infection. The allele CC genotype has been related to higher levels of some drugs (Zhu et al., 2014), and we found that the CC genotype was associated with an increased risk of infections, so this could be due to higher plasmatic levels of antineoplastic and, therefore, a higher probability of adverse events.

### The Usefulness of the Model

We can calculate the probability of having an infection by combining all factors and coefficients in the model we developed. Nevertheless, the clinical action taken due to the calculated value should be consistent with the clinical objective. An important issue is determining the output probability over which the patient will be considered as a probable case or not. Depending on the probability cutoff selected, the model’s sensibility and specificity would change, and it is relevant to ponder which indicator will be better for clinical outcomes, workflows, and resources.

**FIGURE 3:**
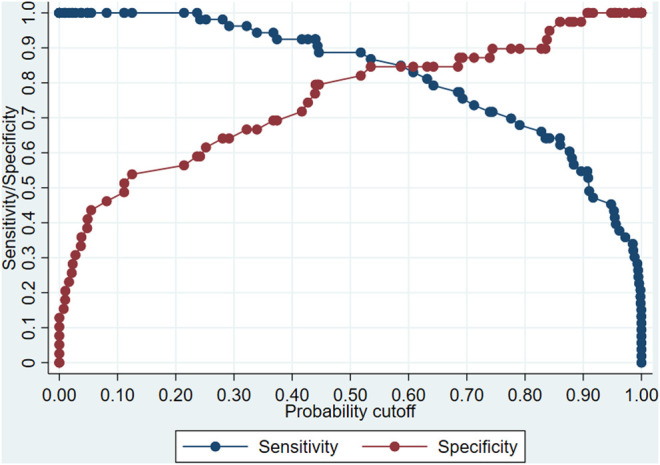
Change in sensitivity and specificity of the model proposed to predict infections in patients undergoing cytotoxic chemotherapy according to the cutoff point for the definition of case.

Figure 3 shows the change of sensitivity (blue line) and specificity (red line) of the proposed model according to different cutoffs. If a balance between sensitivity and specificity is sought, a better cutoff point would be 0.6 (that is, above 0.6, the patient would be classified as having an infection and under the value, as without infection). This cutoff is the intersection point of curves, where both specificity and sensitivity are in values around 85%.

If it is more important that all or a large part of the possible cases are correctly classified, sensitivity should be privileged, and a lower probability cutoff should be chosen. For example, with a cutoff point of 0.3, the sensitivity is 100%, which ensures that most cases (true positives) are detected, with the disadvantage that some patients who will not suffer an infection will be misclassified (false positives). In this example, the model could be used to give medical discharge to those patients who have a low risk or probability of infection.

On the other hand, if it is sought to correctly classify all patients (or the vast majority) who will not suffer an infection (true negatives), a higher cutoff point should be set. For example, by setting a cutoff point of 0.9, we ensure that no patient (or very few) who will not suffer an infection are classified as a case. The disadvantage of this approach is that the rate of those classified as non-cases would increase, even though they will present an infection (false negatives). In this example, the model could help patients at high risk of infection be referred to an isolation room or receive optimized antimicrobial prophylaxis from the start of chemotherapy since the model indicates the patient has a high probability of suffering an infection.

Concerning the performance of the proposed model, the value of the AUC-ROC is high. With an AUC-ROC value over 0.8, the model makes a proper classification of people who will have the event and who will not (Harrell, 2015). The Hosmer–Lemeshow test *p*-value indicates that the frequency of cases predicted by the model was similar to our sample’s observed values.

Several studies have tried to predict the incidence of infections in patients with hematological malignancies, but no one has combined genetic and clinical factors in a single model. Webb et al. created an algorithm to predict the incidence of bloodstream infections due to vancomycin-resistant *enterococcus* in patients undergoing leukemia induction and included severe neutropenia as one of the factors adding to the use of some antimicrobials previously; they found an AUC-ROC curve of 0.84 (Webb et al., 2017).

Schalk et al. used a modified Infection Probability Score (mIPS) to predict the incidence of central venous catheter-related bloodstream infections in patients with hematological malignancies. The mIPS includes clinical variables such as the heart and respiratory rates and also the WBC count. They compared the patients’ score at the moment of catheter insertion and removal; the AUC-ROC curve was 0.77 (Schalk et al., 2015). Apostolopoulou et al. included in their model the presence and length of neutropenia, and similarly to what we found with longer neutropenia associated with more risk of infection, they also identified chemotherapy as a risk factor (Apostolopoulou et al., 2010).

### Non-Genetic Factors Associated With the Risk of Infections

Other risk factors we identified as associated with the risk of infection were acute lymphoblastic leukemia diagnosis compared to acute myeloblastic leukemia and the number of days in profound neutropenia. The first factor could be due to differences in treatment and even the illness’s pathophysiology (Sung et al., 2007; Chandran et al., 2012; Inaba et al., 2017). An absolute neutrophil count lower than 500 cells/mm3 is one of the more significant factors associated with infection, so the longer neutropenia lasts, the higher the risk (Gil et al., 2007). Male sex seems to be a protective factor. It could be because women are more susceptible to hematological toxicities during chemotherapy, increasing the risk of infection (Singh et al., 2005), making them more likely to have a bloodstream infection with hematology-oncology illness (Apostolopoulou et al., 2010). Additionally, women often have smaller body size than men and may be more likely to experience chemotoxicity.

It is necessary to consider non-genetic variables in the analysis because they can explain the intraindividual variability of infection, having genetics unchanged. Differences could be attributed to changes in the chemotherapy scheme or doses, different use of G-CSF or prophylaxis, and other unmeasured variables as food, visits, or bed availability. With this model, we can better understand some of the factors (genetic or not) involved in chemotherapy response.

### Limitations

This study aimed to create a preliminary algorithm and identify genetic and clinical variables associated with infection risk in patients undergoing chemotherapy. Nevertheless, these results could be used as a base for new studies. Because of the small sample size, we could not split our cohort into a training and test cohort for the model development. As a result, the performance metrics of our prediction model likely reflect a degree of overfitting. The prediction algorithm should not be directly applied to clinical practice. Future studies are needed to validate the model and set cutoff decision parameters to improve clinical care and outcomes.

One of the proposed model’s main limitations is that it uses the number of days in profound neutropenia as an explanatory variable and other data obtained directly from the clinical record. However, if we want to use this prediction model at the beginning of the treatment cycle, we should use the expected number of days, causing the model to lose accuracy because it would be an approximate number, not the actual length of neutropenia. A solution to this problem is to generate a second model to predict neutropenia duration and feed its results into the previous model to predict infection risk.

Due to the sample size, we decided not to incorporate some pharmacotherapeutic variables, mainly the specific chemotherapy scheme or the use of granulocyte colony-stimulating factors. In a larger sample, these variables should be included to improve the precision of the model. Besides, other possible not measured explanatory variables, such as the severity of the illness or the gut microbiome, may influence infection risk (Hakim et al., 2018).

The sample size should be analyzed in the context of a developing way country, with a population of 18 million and an incidence of hematological malignancies of 15 cases per 100,000 habitants/year. Also, the prospective character of the study makes the rate of recruitment similar to the incidence rate. This sample size means almost all the new cases in a year in two hospitals in Chile. Although we did not reach a power to discard other polymorphisms, we got the confidence of four polymorphism were related to the event.

## Conclusion

*CYP3A4* rs2242480C>T, *SLC22A11* (OAT4) rs11231809T>A, *TLR2* rs4696480T>A, and *IL6* rs1800796C>G genetic polymorphisms are associated with the incidence of infections among patients undergoing cytotoxic chemotherapy. Including these genetic variables with clinical variables leads to a useful prediction tool. This study is the first to use genetic variables in addition to clinical variables to predict the incidence of infections in patients with hematological malignancies undergoing cytotoxic chemotherapy and could lead to improved clinical outcomes for patients.

## Data Availability

The datasets presented in this study can be found on doi.org/10.6084/m9.figshare.13444211. The names of the repository/repositories and accession number(s) can be found in the article/ Supplementary Material.
