# Supplementary material for: Pharmacogenetics–Based Preliminary Algorithm to Predict the Incidence of Infection in Patients Receiving Cytotoxic Chemotherapy for Hematological Malignancies: A Discovery Cohort
Source: Front Pharmacol. 2021 Mar 10;12:602676. doi: 10.3389/fphar.2021.602676 (PMC7988592; doi:10.3389/fphar.2021.602676)
Supplement: Supplementary file 1 [file table1.docx]

Supplementary Table S1. Uni-genic multivariate analysis for the relationship between genetic polymorphism and the incidence of infection.

|  |  |  | |  |
| --- | --- | --- | --- | --- |
| Variable | Odds Ratio | Confidence Interval 95% | | p value |
| CYP3A4 CC | 5,74 | 1,56 | 21,18 | 0,009 |
| ABCC2 GG | 5,69 | 1,10 | 29,39 | 0,038 |
| OAT4 TT | 0,02 | 0,002 | 0,21 | 0,001 |
| OAT4 AT | 0,08 | 0,01 | 0,54 | 0,009 |
| TLR2 TT | 4,23 | 1,04 | 17,15 | 0,044 |
| IL6 CC/CG | 5,23 | 1,09 | 25,20 | 0,039 |
|  |  |  |  |  |

All these analyses were controlled by cycle of chemotherapy, type of chemotherapy, age, diagnosis and days in severe neutropenia.
